# Delayed CO_2_ postconditioning promotes neurological recovery after cryogenic traumatic brain injury by downregulating IRF7 expression

**DOI:** 10.1111/cns.14268

**Published:** 2023-05-19

**Authors:** Yan Li, Ru Chen, Gui‐Ping Shen, Jing Yin, Yu Li, Jing Zhao, Fang Nan, Shu‐Han Zhang, Hui‐Feng Zhang, Cai‐Hong Yang, Mei‐Na Wu, Yan‐Ying Fan

**Affiliations:** ^1^ Department of Pharmacology, Basic Medical Sciences Center Shanxi Medical University Taiyuan China; ^2^ Key Laboratory of Cellular Physiology, Ministry of Education Shanxi Medical University Taiyuan China

**Keywords:** brain tissue repair, delayed CO_2_ postconditioning, interferon regulatory factor 7, traumatic brain injury

## Abstract

**Aims:**

Few treatments are available in the subacute phase of traumatic brain injury (TBI) except rehabilitation training. We previously reported that transient CO_2_ inhalation applied within minutes after reperfusion has neuroprotective effects against cerebral ischemia/reperfusion injury. In this study, it was hypothesized that delayed CO_2_ postconditioning (DCPC) starting at the subacute phase may promote neurological recovery of TBI.

**Methods:**

Using a cryogenic TBI (cTBI) model, mice received DCPC daily by inhaling 5%/10%/20% CO_2_ for various time‐courses (one/two/three cycles of 10‐min inhalation/10‐min break) at Days 3–7, 3–14 or 7–18 after cTBI. Beam walking and gait tests were used to assess the effect of DCPC. Lesion size, expression of GAP‐43 and synaptophysin, amoeboid microglia number and glia scar area were detected. Transcriptome and recombinant interferon regulatory factor 7 (*Irf7*) adeno‐associated virus were applied to investigate the molecular mechanisms.

**Results:**

DCPC significantly promoted recovery of motor function in a concentration and time‐course dependent manner with a wide therapeutic time window of at least 7 days after cTBI. The beneficial effects of DCPC were blocked by intracerebroventricular injection of NaHCO_3_. DCPC also increased puncta density of GAP‐43 and synaptophysin, and reduced amoeboid microglia number and glial scar formation in the cortex surrounding the lesion. Transcriptome analysis showed many inflammation‐related genes and pathways were altered by DCPC, and *Irf7* was a hub gene, while overexpression of IRF7 blocked the motor function improvement of DCPC.

**Conclusions:**

We first showed that DCPC promoted functional recovery and brain tissue repair, which opens a new therapeutic time window of postconditioning for TBI. Inhibition of IRF7 is a key molecular mechanism for the beneficial effects of DCPC, and IRF7 may be a potential therapeutic target for rehabilitation after TBI.

## Graphical abstract

Delayed CO_2_ postconditioning (DCPC) promoted motor functional recovery accompanied by reduced glial scar formation and microglial activation, and enhanced axon regeneration after cryogenic traumatic brain injury (TBI), with a wide therapeutic time window. Inhibition of neuroinflammation by downregulating the expression of IRF7 is a key molecular mechanism for DCPC to perform beneficial effects. DCPC or targeting IRF7 may be a new intervention for rehabilitation of TBI patients.

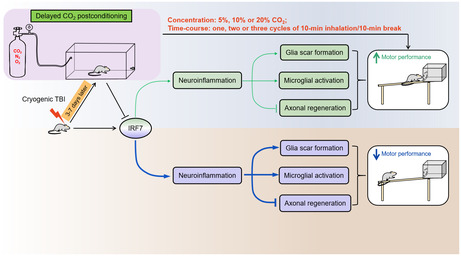

## 1 INTRODUCTION

Traumatic brain injury (TBI) is a disease with high mortality and disability. In addition to primary injury caused by external forces, TBI often progresses to a secondary injury including inflammatory responses, oxidative stress, and blood–brain barrier permeability^1^, ^2^, ^3^ which can lead to neurological deficits. Therefore, most survivors of TBI experience long‐term disabling changes in cognition, sensorimotor function, and personality.^4^ Currently, rehabilitation training is the main therapy available for the enduring deficits induced by TBI,^5^ which may enhance a large amount of endogenous repair processes such as neurogenesis, synaptogenesis, axonal remodeling, and angiogenesis after TBI.^6^, ^7^, ^8^ However, rehabilitation training takes a long time to complete and has limited benefits.^9^ There is an urgent need to explore more effective therapies for the treatment of TBI.

Postconditioning is a promising neuroprotective strategy against cerebral ischemia/reperfusion injuries,^10^, ^11^ which is carried out by giving a sublethal stimulus such as transient cerebral ischemia, hypoxia, and limb remote ischemia^12^, ^13^, ^14^, ^15^ to mobilize the endogenous protective or reparative mechanisms of brain. Although cerebral ischemia and TBI have different pathological bases, they share some common secondary injury processes. It seems reasonable that some treatments for cerebral ischemia may be equally effective for TBI. In recent years, preclinical and clinical studies have indicated that remote ischemia postconditioning plays a neuroprotective role in TBI as well as cerebral ischemia/reperfusion injury.^14^, ^16^ Single or continuous remote ischemic postconditioning starting at 2 h after TBI decreased cognitive and motor deficits in a mouse model.^16^, ^17^ The clinical trials showed that remote ischemic postconditioning given to TBI patients immediately after admission enhanced neurological recovery and decreased the biomarkers of acute brain injury in patients, such as S‐100B and NSE.^18^, ^19^ However, to our knowledge, existing studies have delivered postconditioning within hours post‐TBI. It remains unclear whether delayed daily postconditioning starting at the subacute phase (3 days to weeks post‐injury) can be applied as a novel treatment for rehabilitation after TBI.

We previously found that postconditioning by transient inhalation of CO_2_ within a few minutes after focal cerebral ischemia–reperfusion reduced the lesion size via simulating the ischemic postconditioning‐induced weak acidic environment in brains of mice and inhibiting mitochondrial‐dependent apoptosis.^20^ Shen et al. further indicated that CO_2_ postconditioning extended the thrombolytic time window for stroke therapy by enhancing ischemia–reperfusion‐induced mitophagy.^21^, ^22^ Considering that inhaling CO_2_ is easy to perform, noninvasive and low cost, it may be a very promising endogenous neuroprotective strategy for clinical transformation.^23^ However, the effects of delayed CO_2_ postconditioning (DCPC) on rehabilitation after TBI have not been reported thus far. We hypothesized that DCPC may promote neurological recovery and brain tissue repair after TBI.

## 2 METHODS

### 2.1 Animals

Male C57BL/6 mice aged 8–9 weeks and weighing 22–24 g were purchased from the Animal Experiment Center of Shanxi Medical University. All animals were kept at 22–24°C and housed under a regimen of 12 h light/12 h dark cycles with free access to food and water. All procedures on animals were planned and conducted according to the ARRIVE guidelines, conformed to the U.S. Public Health Service Policy on Humane Care and Use of Laboratory Animals, and approved by the Institutional Animal Care and Use Committee at Shanxi Medical University (Approval Number: SYDL2022011). All the experimental groups were randomized and the experimenters were blinded to the treatment condition.

### 2.2 Cryogenic TBI (cTBI) model

The cTBI model was constructed as described previously.^24^, ^25^ Detailed experimental procedures are shown in the Appendix S1.

### 2.3 Protocols of DCPC

**FIGURE 1:**
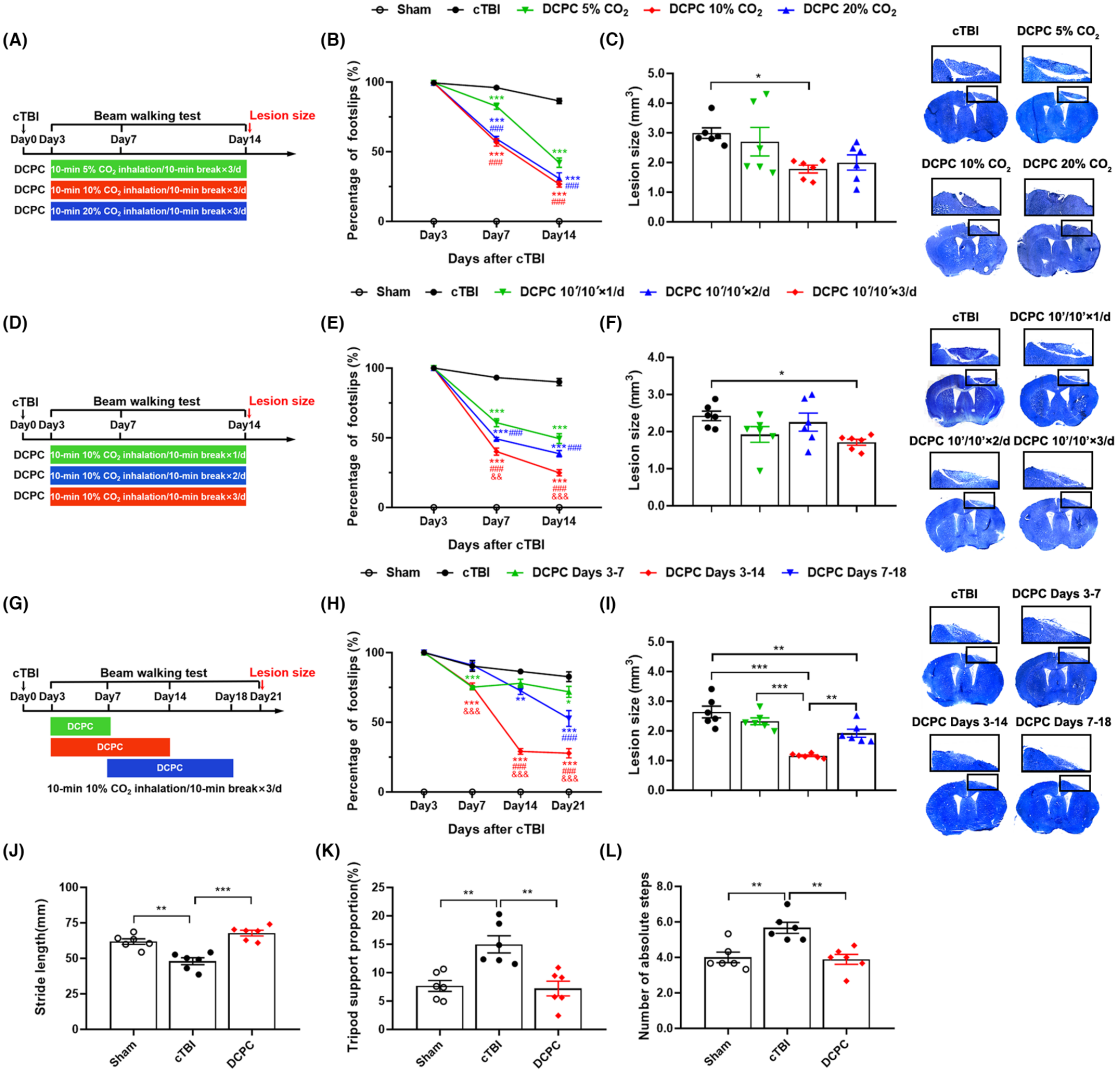
Effects of DCPC on motor function outcomes and lesion size after cTBI. (A) Diagram showing the design of the experiments in B‐C. (B) Percentage of footslips in the left hindlimbs of different concentrations of DCPC, ***p < 0.001 vs cTBI group, ###*p* < 0.001 vs 5% CO_2_ group. (C) The effect of different concentrations of DCPC on lesion size at Day 14 post‐injury, the right panel showed the representative coronal brain sections for lesion size, *p < 0.05 vs cTBI group. (D) Diagram showing the design of the experiments in E‐F. (E) Percentage of footslips in the left hindlimbs of different time courses of DCPC, ***p < 0.001 vs cTBI group, ###*p* < 0.001 vs 10′/10′ × 1/day group, &&*p* < 0.01, &&&*p* < 0.001 vs 10′/10′ × 2/day group. (F) The effect of different time courses of DCPC on lesion size at Day 14 post‐injury, the right panel showed the representative coronal brain sections for lesion size, *p < 0.05 vs cTBI group. (G) Diagram showing the design of the experiments in H‐I. (H) Percentage of footslips in the left hindlimbs of different treatment time windows of DCPC, **p < 0.01, ***p < 0.001 vs cTBI group, ###*p* < 0.001 vs Days 3–7 group, &&&*p* < 0.001 vs Days 7–18 group. (I) The effect of different treatment time windows of DCPC on lesion size at Day 21 post‐injury, the right panel showed the representative coronal brain sections for lesion size, **p < 0.01, ***p < 0.001. (J)‐(L) Effect of DCPC (10% CO_2_, 10′/10′ × 3/day, administered at Days 3–14 after cTBI) on gait performance including stride length (J), tripod support proportion (K) and the number of absolute steps (L) in the left limbs, **p < 0.01, ***p < 0.001 vs cTBI group. Two‐way ANOVA with Tukey's test was used for analysis in B, E and H. One‐way ANOVA with Tukey's test was used for analysis in C, F, I, J, K and L. The results are shown as the mean ± SEM, *n* = 6. 10′ /10′: 10‐min CO_2_ inhalation/10‐min break.

DCPC was administered to the animals by making them inhale CO_2_ (21% O_2_ supplemented with N_2_) for 10 min with a flow rate of 1 L/min and resting for 10 min as one cycle per day (10′/10′ × 1/day) in an airtight chamber (1100 mL). The control mice received the same procedure with atmospheric air inhalation. In the concentration‐dependent experiment, mice were treated with three cycles of DCPC by inhaling 5%, 10% or 20% daily during Days 3–14 after injury (Figure 1A). In the time‐course‐dependent experiment, the treated mice were given one cycle (10′/10′ × 1/day), two cycles (10′/10′ × 2/day) or three cycles (10′/10′ × 3/day) of DCPC with 10% CO_2_ during Days 3–14 after injury (Figure 1D). In the time‐window experiment, DCPC (10% CO_2_, 10′/10′ × 3/day) was administered from Days 3–7, 3–14, or 7–18 after injury (Figure 1G). In the NaHCO_3_ reverse experiment, NaHCO_3_ (2 μL/animal, 78 mg/mL) was injected into the lateral ventricles of the mice followed by DCPC (10% CO_2_, 10′/10′ × 3/day) which was administered daily during Days 3–14 after cTBI.

### 2.4 Injection of adeno‐associated virus (AAVs)

Recombinant interferon regulatory factor 7 (*Irf7*) AAVs (AAV‐*Irf7*, HBAAV2/9‐CMV‐*Irf7*‐3flag‐ZsGreen) and empty AAVs (AAV‐Con, HBAAV2/9‐CMV‐ZsGreen) were designed and produced by Hanbio Biotechnology (Shanghai, China). Detailed experimental procedures are shown in the Appendix S1.

### 2.5 Beam walking test

Beam walking test was conducted as described before.^24^, ^25^ Detailed experimental procedures are shown in Appendix S1.

### 2.6 Gait test

Gait test was conducted as described before.^25^ Detailed experimental procedures are shown in Appendix S1.

### 2.7 Measurement of the lesion size

Detailed experimental procedures are shown in Appendix S1.

### 2.8 Immunofluorescence

The immunofluorescence staining was conducted as described before.^24^ Detailed experimental procedures are shown in Appendix S1.

### 2.9 Transcriptome analysis

Total mRNA was extracted using TRIzol. Detailed data acquisition and analysis procedures are provided in Appendix S1.

### 2.10 Quantitative Real‐Time PCR

Total mRNA was extracted using TRIzol. Detailed experimental procedures are shown in Appendix S1. The primer sequences are listed in Appendix S1: Table S1.

### 2.11 Western Blot analysis

Detailed experimental procedures are shown in Appendix S1.

### 2.12 Statistical analysis

All data were collected in a blinded manner and analyzed using GraphPad Prism 8 software. The mean ± SEM values are presented. All data were tested by Shapiro–Wilk normality test. The comparisons between two groups with one independent factor were analyzed using an unpaired t test. One‐way ANOVA followed by Tukey's multiple comparisons test was utilized to compare three or more groups with one independent factor. Two‐way ANOVA followed by Tukey's multiple comparisons test was utilized to perform two independent variable analyses. *p* value <0.05 was considered statistically significant.

## 3 RESULTS

### 3.1 DCPC promoted motor function recovery and reduced brain lesion size in mice after cTBI

To investigate the optimal concentration of DCPC for improving neurological dysfunction, mice inhaled 5%, 10% or 20% CO_2_ (10′/10′ × 3/day) from Days 3–14 after cTBI (Figure 1A). The footslips percentage of mice in all DCPC groups was significantly lower than that in the cTBI group at Day 7 and Day 14 and the effect of 10% CO_2_ group was the best (*p* < 0.001 vs cTBI group, Figure 1B). In addition, 10% CO_2_ DCPC significantly reduced lesion size, while DCPC with 5% and 20% CO_2_ displayed no significant effect (*p* < 0.05 vs cTBI group, Figure 1C).

Next, we explored the time course of DCPC (Figure 1D). The results showed that DCPC with 10% CO_2_ inhalation of 10′/10′ × 2/day and 10′/10′ × 3/day was more effective than 10′/10′ × 1/day in improving motor function after cTBI (*p* < 0.001 vs 10′/10′ × 1/day group, Figure 1E). As three cycles of inhalation daily possessed the best effect on decreasing cerebral lesion size (*p* < 0.05 vs cTBI group, Figure 1F), mice were given this treatment regimen to explore the optimal treatment time window.

Then, we explored the critical period and therapeutic time window of DCPC (Figure 1G). DCPC administered at Days 3–7, Days 3–14 or Days 7–18 after cTBI significantly reduced the footslips percentage of mice (*p* < 0.05, *p* < 0.01, *p* < 0.001 vs cTBI group, Figure 1H). The improvement effect of the Days 3–7 group was significantly weaker than that of the Days 3–14 group at Day 14 and Day 21 (*p* < 0.001 vs Days 3–7 group, Figure 1H), which indicated that the cumulative effect of continuous 10% CO_2_ inhalation was necessary for its therapeutic efficacy. Although the improvement of motor function by DCPC administered at Days 7–18 after cTBI was weaker than that at Days 3‐14, we found that even the inhalation of 10% CO_2_ starting at Day 7 after cTBI could still decrease the footslips percentage by almost 30% compared to the cTBI group (*p* < 0.01, *p* < 0.001 vs cTBI group, Figure 1H) and reduce cerebral lesion size (*p* < 0.01 vs cTBI group, Figure 1I), which means that the therapeutic effect of DCPC on cTBI may have a wide time window. Since the general performance of Days 3–14 group with 10% CO_2_ (10′/10′ × 3/day) was the best among the DCPC groups, this condition was used in the subsequent experiments.

In addition, the gait test (Figure 1J‐L) showed that DCPC (10% CO_2_, 10′/10′ × 3/day) administered at Days 3–14 reversed the increase in the tripod support proportion (*p* < 0.01 vs cTBI group, Figure 1K) and the absolute steps (*p* < 0.01 vs cTBI group, Figure 1L) and the decrease in the stride length (*p* < 0.01, *p* < 0.001 vs cTBI group, Figure 1J) caused by cTBI at Day 14.

### 3.2 The effects of DCPC on the motor functional recovery and cerebral lesion size after cTBI were reversed by NaHCO_3_

To investigate whether the effect of DCPC on neurological recovery after cTBI was related to the acidification of brain tissue induced by CO_2_ inhalation, the mice were intracerebroventricularly administered NaHCO_3_. The beneficial effects of DCPC on neurological function and lesion size were reversed by combined application with NaHCO_3_ (*p* < 0.01, *p* < 0.001 vs cTBI group, *p* < 0.01, *P* < 0.001 vs NaHCO_3_ + DCPC group, Appendix S1: Figure S1A, B), while NaHCO_3_ alone had no obvious effects (Appendix S1: Figure S1A, B).

### 3.3 DCPC enhanced synaptic plasticity, and inhibited microglial overactivation and glial scar formation after cTBI

**FIGURE 2:**
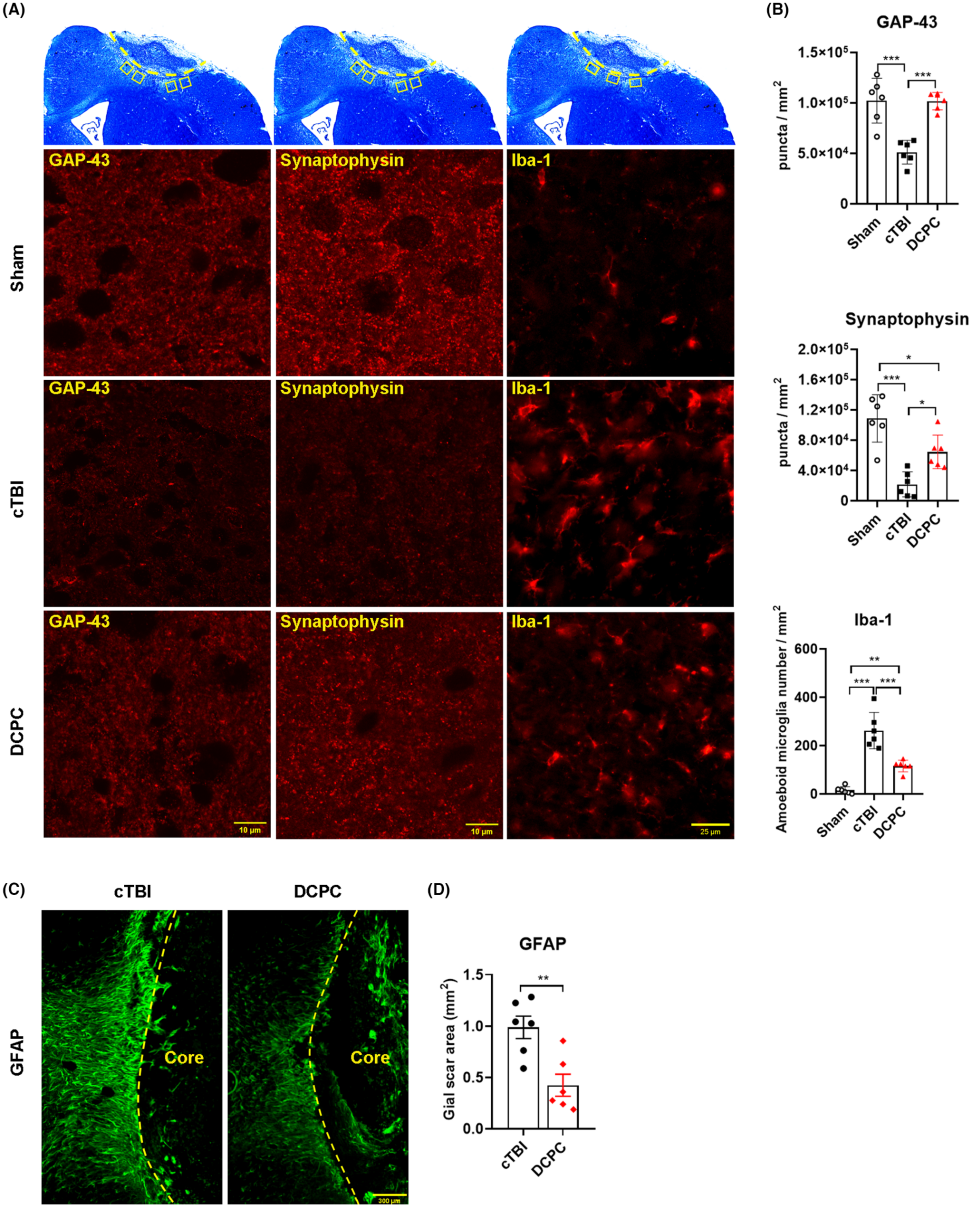
Effect of DCPC on brain tissue repair processes, microglial activation and glial scar formation post‐injury. (A) Representative GAP‐43, synaptophysin and Iba‐1 immunofluorescence images of the cortex surrounding the lesion in mice, the upper panel showing the photographic range of each index. (B) The puncta density of GAP‐43 and synaptophysin and the number of Iba‐1‐positive amoeboid microglia in the cortex surrounding the lesion in mice, *p < 0.05, **p < 0.01, ***p < 0.001. (C) Representative images of glial scars with GFAP immunofluorescence staining in mice. (D) Glial scar area of the cortex surrounding the lesion in mice, **p < 0.01. One‐way ANOVA with Tukey's test was used for analysis in B. Unpaired t test was used for analysis in D. The results are shown as the mean ± SEM, *n* = 6.

To determine whether DCPC promoted axonal regeneration and synaptogenesis, we detected the expression of GAP‐43 and synaptophysin (Figure 2A). The puncta densities of GAP‐43 and synaptophysin in the cortex surrounding the lesion decreased significantly at Day 14 after cTBI, while these adverse effects were attenuated by DCPC (*p* < 0.05, *p* < 0.001 vs cTBI group, Figure 2A, B). Furthermore, the number of Iba‐1‐positive amoeboid microglia in the cortex surrounding the lesion (*p* < 0.001 vs cTBI group, Figure 2A, B) and the glial scar area assessed by GFAP staining (*p* < 0.01 vs cTBI group, Figure 2C, D) decreased dramatically in the DCPC group compared to the cTBI group at Day 14 post‐injury.

### 3.4 Transcriptome analysis showed that DCPC significantly altered genes expression in the cortex tissue surrounding the lesion in mice

**FIGURE 3:**
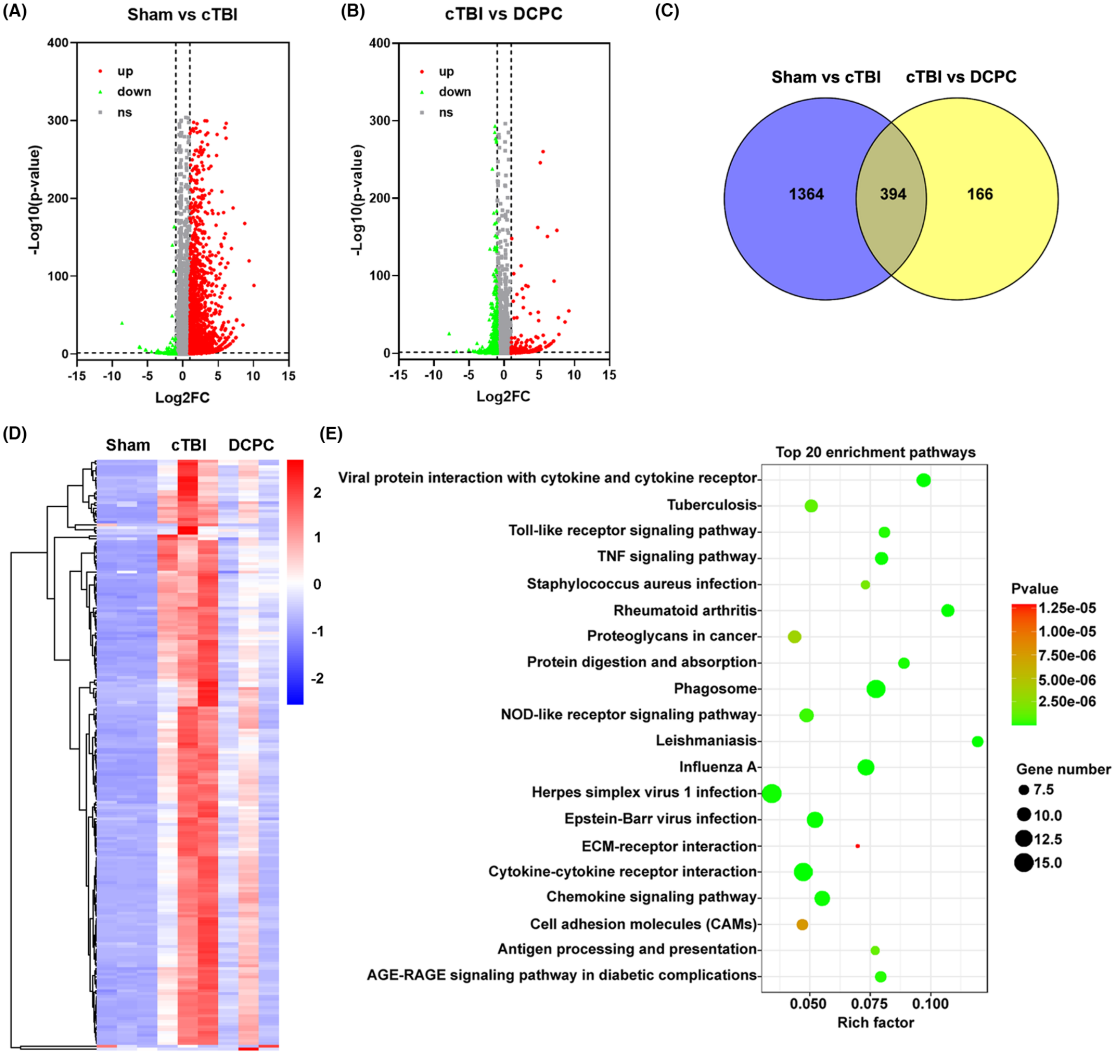
Transcriptome analysis of the cortex tissue surrounding the lesion in mice in the Sham, cTBI and DCPC groups. (A) The volcano map of Sham vs cTBI group. Red dots indicate genes with high levels of expression, green dots indicate genes with low levels of expression, and gray dots indicate genes with no differential expression based on the criteria of *p* value <0.01 and |log2FoldChange|>1. (B) The volcano map of cTBI vs DCPC group. Red dots indicate genes with high levels of expression, green dots indicate genes with low levels of expression, and gray with no differential expression based on the criteria of *p* value <0.01 and |log2FoldChange|>1. (C) Venn diagram of 394 DEGs from the microarray datasets of Sham vs cTBI group and cTBI vs DCPC group. (D) Heatmap of 213 DEGs differentially expressed in the Sham, cTBI and DCPC groups. Gene expression levels are indicated by colors as shown by the row; red represents a high expression level, and blue represents a low expression level. (E) The top 20 KEGG enrichment pathways of DEGs. *n* = 3.

**FIGURE 4:**
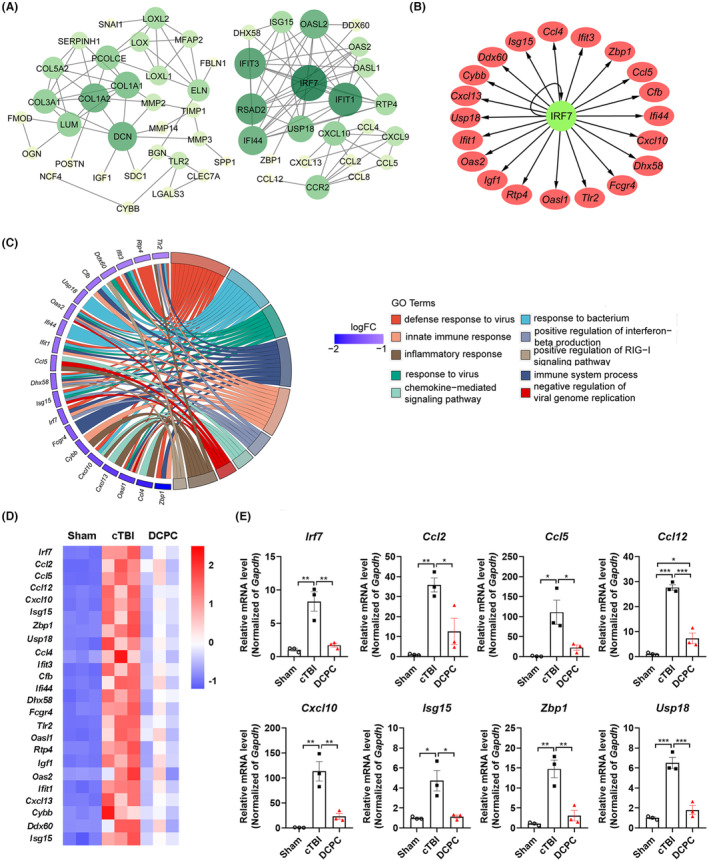
Enrichment analysis of DEGs in the transcriptome and validation of transcriptome genes expression using qPCR. (A) PPI network analysis. The color and size represent the degree of nodes. (B) The predicted downstream target genes of IRF7. The green circle node represents IRF7, and the red circle node represents the downstream target genes of IRF7. (C) GO term analysis of IRF7‐regulating genes among DEGs. (D) Transcriptome results of downstream target and related genes of IRF7. (E) The mRNA expression of *Irf7*, *Ccl2*, *Ccl5*, *Ccl12*, *Cxcl10*, *Isg15*, *Zbp1* and *Usp18* in the cortex tissue surrounding the lesion in mice in the Sham, cTBI and DCPC groups, * *p* < 0.05, **p < 0.01, ***p < 0.001. One‐way ANOVA with Tukey's test was used for analysis. The results are shown as the mean ± SEM, *n* = 3.

To investigate the molecular mechanism of DCPC, tissue samples were collected for transcriptome analysis. The expression of 1758 genes were altered remarkably after cTBI (Figure 3A) and 560 genes were changed by DCPC treatment compared with the cTBI group (Figure 3B). The Venn diagram showed that 394 out of the 560 differentially expressed genes (DEGs) responding to DCPC were related to cTBI (Figure 3C) and the hierarchical clustering showed that DCPC significantly reversed the expression of genes altered by cTBI (Figure 3D). In addition, qPCR assays were performed to assess the reliability of the transcriptome data (Figure 4D‐E). The expression of altered genes such as *Irf7*, C‐C motif ligand 2 (*Ccl2*), C‐C motif ligand 5 (*Ccl5*), C‐C motif ligand 12 (*Ccl12*), C‐X‐C motif chemokine ligand 10 (*Cxcl10*), ISG15 ubiquitin‐like modifier (*Isg15*), Z‐DNA binding protein 1 (*Zbp1*), and ubiquitin specific peptidase 18 (*Usp18*) was significantly decreased by DCPC (*p* < 0.05, *p* < 0.01, *p* < 0.001 vs cTBI group, Figure 4E), consistent with the transcriptomic data.

To determine the signaling pathways in which the DEGs participated, Kyoto Encyclopedia of Genes and Genomes (KEGG) pathway analysis was performed. The results suggested that the significantly enriched pathways mainly included cytokine–cytokine receptor interaction, tumor necrosis factor (TNF) signaling pathway, and chemokine signaling pathway, among others (Figure 3E).

### 3.5 IRF7 was a master transcription factor in DEGs regulated by DCPC

To investigate biomolecular interaction networks and identify key proteins among the DEGs, the protein–protein interaction (PPI) network was performed and the results indicated that the principal relevant molecule was IRF7 (Figure 4A). Some of DEGs may be potential downstream target genes of IRF7 such as toll‐like receptor 2 (*Tlr2*), *Ccl5*, *Cxcl10*, C‐X‐C motif chemokine ligand 13 (*Cxcl13*), and *Isg15*, among others (Figure 4B). Gene Ontology (GO) enrichment analysis indicated that the IRF7 regulating genes were primarily associated with the inflammatory response, chemokine‐mediated signaling pathway, and immune system response, among others (Figure 4C).

**FIGURE 5:**
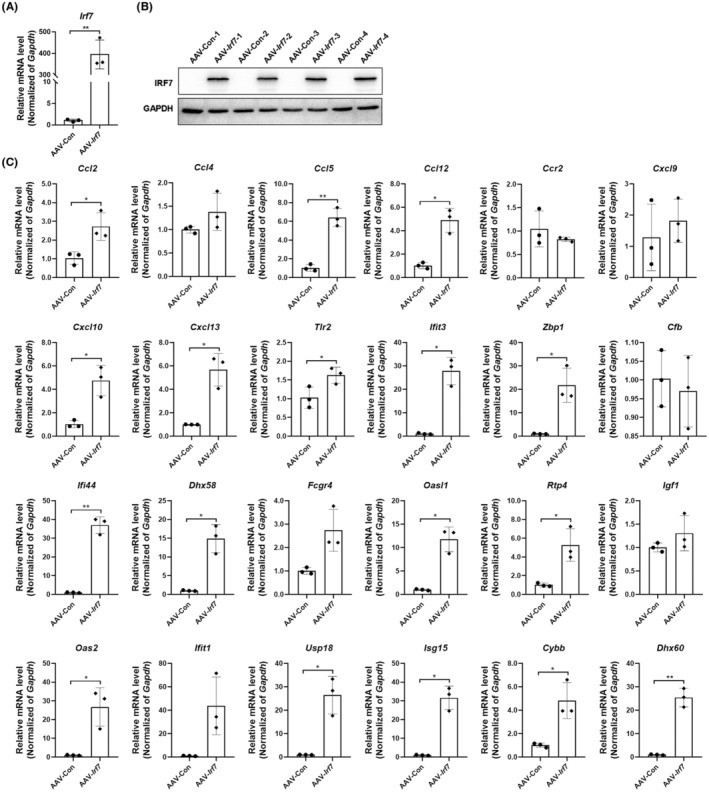
Effects of IRF7 overexpression on the mRNA levels of IRF7 downstream target or relative genes. The AAV‐*Irf7* and AAV‐Con were stereotactically injected into the three points of cortical areas and the tissue samples were acquired at 5 weeks after injection. (A) The *Irf7* mRNA expression in the virus‐injected cortex in mice in the AAV‐Con and AAV‐*Irf7* groups, **p < 0.01 vs AAV‐Con group. (B) The IRF7 protein expression in the virus‐injected cortex in mice in the AAV‐Con and AAV‐*Irf7* groups. (C)The mRNA levels of IRF7 downstream target or relative genes in the virus‐injected cortex in mice in the AAV‐Con and AAV‐*Irf7* groups, *p < 0.05, **p < 0.01 vs AAV‐Con group. Unpaired t test was used for analysis. The results are shown as the mean ± SEM, *n* = 3 per group in A and C, and *n* = 4 per group in B.

IRF7 is a multifunctional transcription factor.^26^ We overexpressed IRF7 to explore whether IRF7 regulated the above downstream target or related genes. After AAV‐*Irf7* injection, IRF7 was successfully overexpressed both in the mRNA and protein levels in the virus‐injected cortex of mice (Figure 5A, B), accompanied by remarkable upregulation of many potential downstream target genes or related genes like *Ccl5*, *Cxcl10*, cytochrome b‐245 beta chain (*Cybb*), and *Isg15* (Figure 5C). These results suggest that IRF7 is a central transcription factor of the above DEGs.

### 3.6 IRF7 may be a target of DCPC for its therapeutic effect on cTBI

**FIGURE 6:**
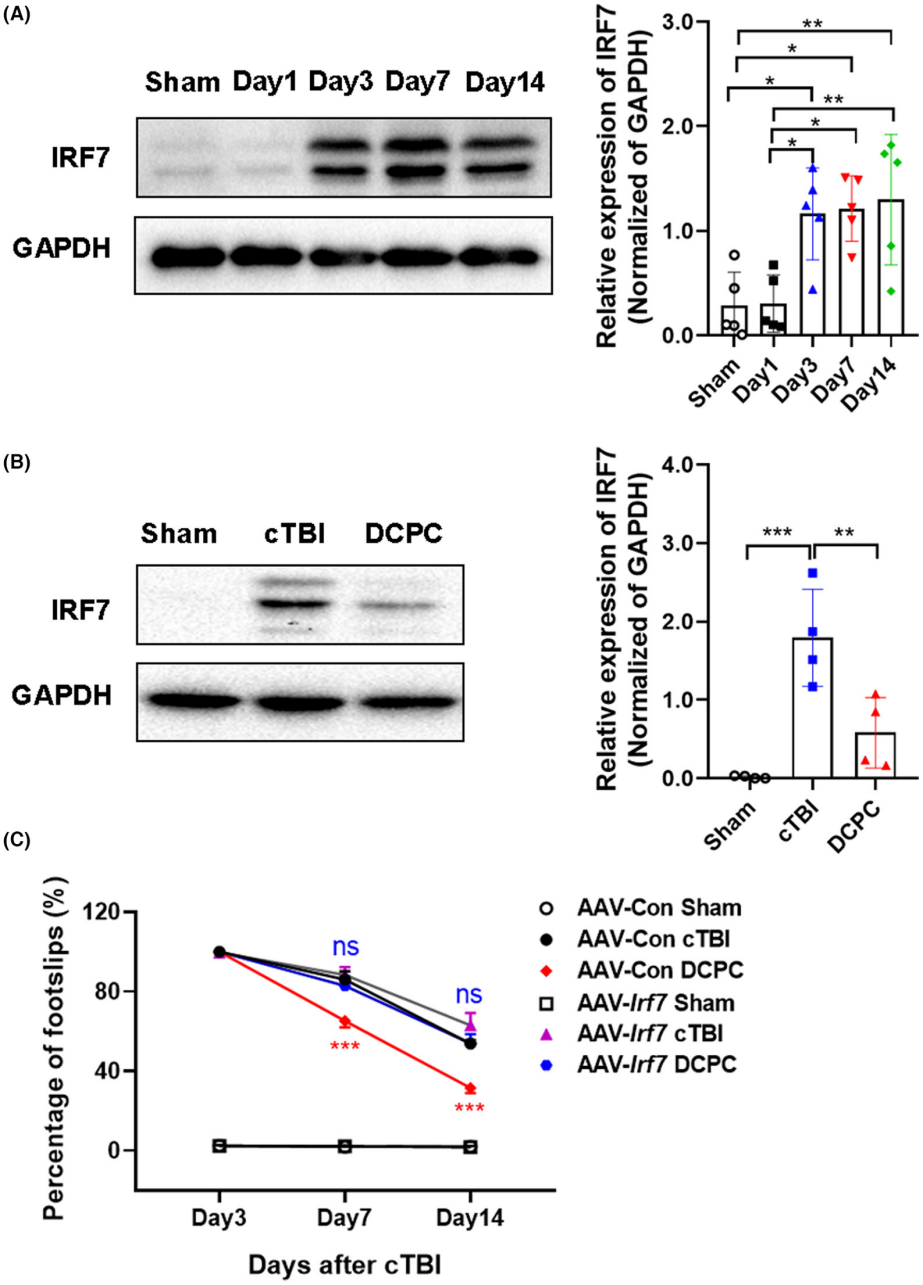
IRF7 is a critical target for the therapeutic effect of DCPC on cTBI. (A) The IRF7 protein expression of the cortex tissue surrounding the lesion in mice at different time points after cTBI. The quantitative results are shown in the right panel, *p < 0.05, **p < 0.01. (B) The IRF7 protein expression in the cortex tissue surrounding the lesion in mice in the Sham, cTBI and DCPC groups. The quantitative results are shown in the right panel, **p < 0.01, ***p < 0.001. (C) Percentage of footslips in the left hindlimbs of mice in the AAV‐*Irf7* or AAV‐Con group that received different treatments, including Sham, cTBI and DCPC (10% CO_2_ inhalation, 10′/10′ × 3/day, administered at Days 3–14 after cTBI), ***p < 0.001 vs AAV‐Con cTBI group. One‐way ANOVA with Tukey's test was used for analysis in A and B. Two‐way ANOVA with Tukey's test was used for analysis in C. The results are shown as the mean ± SEM, *n* = 5 per group in A, *n* = 4 per group in B, and *n* = 9 per group in C.

To investigate the dynamic expression of IRF7 after cTBI, we collected the cortex tissue surrounding the lesion at different time points for western blot analysis. The expression of IRF7 was very low in normal mouse brain tissues, while it increased significantly at Day 3, Day 7 and Day 14 except Day 1 after cTBI (*p* < 0.05, *p* < 0.01 vs Sham group, Figure 6A). DCPC largely inhibited the cTBI‐induced upregulation of IRF7 at Day 14 post‐injury (*p* < 0.01, *p* < 0.001 vs cTBI group, Figure 6B) and the beneficial effect of DCPC was impaired by IRF7 overexpression (ns vs AAV‐*Irf7* cTBI group, Figure 6C). However, DCPC still exerted therapeutic effects in the mice expressing AAV‐Con vector at Day 7 and Day 14 post‐injury (*p* < 0.001 vs AAV‐Con cTBI group, Figure 6C). In conclusion, DCPC may exhibit its pro‐neurological recovery effect by inhibiting IRF7 expression.

## 4 DISCUSSION

We previously found that transient inhalation of CO_2_ in mice within a few minutes after reperfusion produces neuroprotection against cerebral ischemia/reperfusion injury.^20^ Based on this, we showed for the first time that DCPC robustly promoted motor functional recovery and reduced the brain lesion size post‐injury, with a wide therapeutic time window of at least 7 days after cTBI. In addition, in the classical controlled cortical impact (CCI) model, DCPC also promoted neurological recovery at Day 7 and Day 14 post‐injury (Appendix S1: Figure S2). Currently, few delayed postconditioning strategies initiated at the subacute phase of TBI are reported. Therefore, DCPC may be a novel therapeutic strategy for rehabilitation after TBI.

In the present study, DCPC has a wide range of effective concentrations and time courses, since inhaling 5%, 10% or 20% CO_2_ for various time‐courses (10′/10′ × 1/day, 10′/10′ × 2/day or 10′/10′ × 3/day) all significantly improved the recovery of motor function after cTBI. It is worth noting that CO_2_ inhalation has been used in clinical practice. In a pilot study, inhalation of 5% CO_2_ was used to suppress hyperventilation‐induced absence seizures in children.^27^ A study assessing cerebrovascular reactivity by BOLD‐MRI in patients with severe intracranial artery stenosis confirmed no significant side effects after 12 min of 8% CO_2_ inhalation.^28^ A study in men confirmed that 9% CO_2_ inhalation is well tolerated by subjects for up to 10 min without affecting their ability to function coherently.^29^ We also observed that 10% CO_2_ inhalation (10′/10′ × 3/day) for 13 or 14 consecutive days had no obvious effects on heart rate, blood oxygen, respiratory rate and blood pressure, and anxiety‐like behavior (Appendix S1: Figure S3). These human and animal studies demonstrated a favorable risk profile of transient CO_2_ inhalation, which makes it easier for DCPC to translate into clinical trials. Meanwhile, although inhaling 10% CO_2_ for 10 min per day produced improvement on behavior outcomes after cTBI, inhaling CO_2_ (10 min for one cycle) intermittently and repeatedly may produce a larger benefit–risk ratio, which needs for further investigation. In addition, treatment strategies with a wide time window can minimize the damage caused by untimely resuscitation and are therefore more suitable for the rehabilitation of TBI patients. We found that even initiating from Day 7 post‐injury, DCPC (10% CO_2_, 10′/10′ × 3/day) still exhibited a favorable effect on the behavioral outcome of cTBI mice. Considering that inhalation of CO_2_ at the acute phase of TBI may cause cerebral vascular dilation and aggravate bleeding, this delayed paradigm can avoid bleeding risk, which is another advantage of the wide therapeutic time window of DCPC.

Many studies have demonstrated that minutes of CO_2_ inhalation can induce transient and mild acidosis of brain tissue. Inhalation of 10% CO_2_ lowered pH value of the amygdala and the lateral ventricle by ~0.2, and bicarbonate blocked CO_2_‐induced acidosis of brain tissue.^30^ Our previous study also found that inhaling 10% and 20% CO_2_ for 5 min reduced pH value by ~0.16 and ~0.24 in the ischemic cortex of mice, respectively.^20^ Similarly, pH value of blood decreased rapidly by ~0.2 after transient 10% CO_2_ inhalation in men.^29^ In this study, intracerebroventricular injection of bicarbonate largely attenuated the effects of DCPC on motor function and cerebral lesion size. Therefore, brain tissue acidosis induced by CO_2_ inhalation may be a critical factor for DCPC induced‐beneficial effects.

Enhanced synaptic plasticity is believed to play a key role in functional recovery after TBI.^31^, ^32^, ^33^ GAP‐43 is a marker involved in axonal terminal regeneration,^34^ and synaptophysin is a marker involved in synaptogenesis during neuroanatomical remodeling,^35^ these two proteins are related to recovery from TBI.^36^, ^37^, ^38^ In this study, we found that DCPC increased the levels of GAP‐43 and synaptophysin at Day 14 post‐injury, suggesting the contribution of synaptic plasticity induced by DCPC to functional recovery. In addition, in the recovery stage of TBI, over activation of astrocytes and microglia is associated with the inhibition of axonal regeneration and synaptogenesis, and may impede neurological functional recovery.^24^, ^39^, ^40^, ^41^, ^42^ We observed that DCPC decreased Iba‐1 positive amoeboid microglia and the glial scar area at Day 14 post‐injury. Therefore, DCPC may indirectly regulate synaptic plasticity by inhibiting microglial and astrocytic gliosis.

By performing PPI network analysis of DEGs, we found that *Irf7* was the DEG with the largest number of associated genes. The reduction of the mRNA and protein expression of IRF7 induced by DCPC was also verified after cTBI. Numerous studies have demonstrated that IRF7 plays a detrimental role in the progression of brain injury.^43^, ^44^, ^45^ In a CCI model, IRF7 expression increased 2 h post TBI to mount the inflammatory response.^43^ IRF7 was proven to regulate the microglial polarization switch, and knockdown of IRF7 inhibited the mRNA level of the neurotoxic microglial M1 marker.^44^ However, the exact roles of IRF7 during the subacute phase of TBI remain unclear. Our study found that the expression of IRF7 in the normal mouse brain was extremely low while robustly elevated in the cortex surrounding the lesion on Days 3–14 post‐injury. The sustained elevation implies the important role of IRF7 in the subacute phase of cTBI. This at least partially illustrated why application of DCPC on Days 3–7 has a weaker effect on motor function recovery than on Days 3–14 post‐injury; that is, continuous inhibition of IRF7 expression may be required for a better therapeutic effect. It also explained why DCPC, initiating from Day 7 post‐injury, still improved the behavioral outcomes. Moreover, overexpression of IRF7 blocked DCPC‐induced improvement of motor function after cTBI, which further demonstrated that DCPC exerts beneficial effects by downregulating IRF7 expression. IRF7 may be a potential target for the treatment in the subacute phase of TBI. In the future, the exact roles of IRF7 in diverse cell types, such as astrocytes, microglia and neurons at different phases of TBI need to be further investigated.

Furthermore, multiple chemokines such as *Ccl2*, *Ccl5*, *Ccl12*, *Cxcl10*, and *Cxcl13* were upregulated after cTBI and downregulated after DCPC treatment. Numerous studies have shown that many chemokines are pro‐inflammatory factors.^40^, ^46^ For example, the CCL2 and CCL12 and common receptor C‐C motif receptor 2 (CCR2) were substantially and persistently upregulated in the cortex of mice 24 h after TBI^47^ and CCL2 released from astrocytes after spinal cord injury promoted microglial activation and neuronal apoptosis.^48^ The expression of chemokines (e.g., CCL2, CCL5, CXCL10, and CXCL13) was also elevated in peripheral blood after brain injury and strongly associated with poor prognosis of TBI patients.^49^ In our study, these chemokines were predicted to be downstream targets of IRF7 or associated with IRF7 and were significantly upregulated after IRF7 overexpression. We speculated that DCPC may inhibit chemokine‐induced neuroinflammation after TBI through reducing IRF7 expression. In addition, KEGG pathway enrichment showed that DEGs altered by DCPC are mainly involved in a number of signaling pathways related to inflammation, such as the Toll‐like receptor signaling pathway, TNF signaling pathway, cytokine–cytokine receptor interaction, and chemokine signaling pathway. In summary, DCPC may regulate neuroinflammation caused by cTBI.

In addition to chemokines, some other genes associated with neuroinflammation such as *Tlr2* and *Cybb* were predicted downstream target genes of IRF7. TLR2 played an adverse role in the recovery of neurological function after TBI,^50^, ^51^ and it was found to be related to neurotoxic M1‐like microglial activation,^52^, ^53^ glial scar formation,^54^ and impediment of neural regeneration and axonal extension.^55^ CYBB is an enzyme‐producing reactive oxygen species during central nervous system injury^56^ and upregulation of CYBB promotes neurotoxic M1‐like microglial activation.^57^ Inhibiting CYBB reduced oxidative stress and improved functional outcomes following TBI.^56^, ^57^, ^58^ Therefore, inhibition of TLR2 and CYBB expression by downregulating IRF7 may also contribute to DCPC‐induced improvement on brain tissue repair and behavioral outcomes.

## 5 CONCLUSION

In summary, we first showed that DCPC promoted motor functional recovery and brain tissue repair after cTBI with a wide therapeutic time window. Since CO_2_‐inhalation has been used clinically without obvious side effects, DCPC may easily be translated to clinical practice. Furthermore, inhibiting IRF7 expression is a critical molecular mechanism involved in DCPC‐induced neuroreparative effects. This study offers a novel intervention for rehabilitation after TBI, and also provides a theoretical basis for IRF7 to be a potential target for the treatment of TBI at the subacute phase.

## AUTHOR CONTRIBUTIONS

Yan Li, Ru Chen, Gui‐Ping Shen, Jing Yin, Yu Li, Jing Zhao, Fang Nan and Shu‐Han Zhang performed experiments; Yan‐Ying Fan, Yan Li and Mei‐Na Wu designed the experiments; Yan‐Ying Fan and Yan Li wrote the manuscript and prepared the figures; Hui‐Feng Zhang and Cai‐Hong Yang analyzed data; Yan‐Ying Fan and Mei‐Na Wu revised the manuscript; All authors read and approved the final manuscript.

## FUNDING INFORMATION

This work was sponsored by the National Natural Science Foundation of China (grant numbers 81872854, 81202520), and the Fund for Shanxi “1331 Project” Key Subjects Construction.

## CONFLICT OF INTEREST STATEMENT

The authors declare no conflict of interest.

## Supporting information


Appendix S1.

Appendix S2.

Video S1.

## Data Availability

The original data of transcriptome is available in the NCBI database (https://www.ncbi.nlm.nih.gov/bioproject/PRJNA848974). The other data sets are available from the corresponding author upon reasonable request.
