# Supplementary material for: Delayed CO2 postconditioning promotes neurological recovery after cryogenic traumatic brain injury by downregulating IRF7 expression
Source: CNS Neurosci Ther. 2023 May 19;29(11):3378–90. doi: 10.1111/cns.14268 (PMC10580333; doi:10.1111/cns.14268)
Supplement: Supplementary file 1 — Appendix S1. [file CNS-29-3378-s002.docx]

**Supplementary methods**

**1. cTBI model**

Mice were anesthetized with isoflurane and fixed to a stereotaxic apparatus (RWD Life Sciences, China). The scalp was cut along the midsagittal direction to expose the skull. A copper rod (3 mm) precooled with liquid nitrogen was pressed on the skull of mice at 180 g for 21 s (0.5 mm anterior and 2 mm right-lateral to bregma as center). The scalp was sutured, and the mice were placed in an incubator until they woke up. Mice in the sham group underwent the same procedure without liquid nitrogen cooler bar press.

**2. Controlled cortical impact (CCI) model**

Mice were anesthetized with isoflurane and fixed to a stereotaxic apparatus (RWD Life Sciences, China). A right parietal craniotomy (0.5 mm anterior and 2 mm right-lateral to bregma as center) was performed on the mice. The CCI was conducted by a pneumatic CCI device with a 3-mm flat-ended punch (Precision Impactor Device, RWD Life Sciences, China), 1.5 mm displacement of the brain, 4.9 m/s impact velocity, 100 ms impact duration time. After CCI, the skin incision was sealed. The mice were placed in an incubator until they woke up. Mice in sham group were subjected to all aspects of the protocol except for CCI. The results were assessed by investigators who were blinded to the group assignments.

**3. Injection of AAVs**

The AAVs (1.3×10^9^ plaque-forming units/each point) were stereotactically injected into the three points of cortical areas (coordinates: anteroposterior +1.58 mm, mediolateral -2 mm, dorsoventral -1.45 mm; anteroposterior -0.42 mm, mediolateral -2 mm, dorsoventral -1.45 mm; anteroposterior +0.5 mm, mediolateral -1 mm, dorsoventral -1.25 mm). The cTBI model was performed at three weeks after injection.

**4. Beam walking test**

Three days before cTBI, the mice were trained to walk across the balance beam with a diameter of 8 mm, a length of 100 cm and a height of 30 cm without slipping. The balance beam test was used to evaluate the motor function of mice. The sliding rate is equal to the number of sliding steps of the left hindlimb divided by the total number of steps in three repeated trials. If some mice could not pass the balance beam due to serious motor function defects, the sliding rate of these mice was recorded as 100%.

**5. Gait test**

All mice underwent training trials from three days before cTBI. Mice were placed on a glass runway (115 cm long, 6.5 cm wide) and trained to cross the runway freely to a dark box for 20 seconds without any break. At Day 14 after cTBI, Bcam Capture and Runway Scan software was used to capture the paw prints of mice from three repeated trials. Stride length, triple feet support percentage and number of absolute steps of the left forelimb were collected. If the mice stopped, turned, or stagnated in the glass track, the data were not included in the results.

**6. Measurement of the** **lesion size**

At Day 14 or 21 after cTBI, the fixed frozen mouse brain tissues were cut into serial coronal brain sections with a thickness of 20 μm and collected serially at 0.24 mm intervals from bregma +1.18 to -1.22 mm. The slices were stained with 1% toluidine blue and then photographed under a light microscope (Leica, Germany, DM2500). Toluidine blue staining was traced and quantified by ImageJ software. The lesion size was determined based on the following formula: lesion size =∑lesion size of each slice × 0.24 mm.

**7. Immunofluorescence**

Brain tissues were cut into sections with a thickness of 20 μm and incubated with primary antibodies against induction of brown adipocytes 1 (1:500; Novus Biologicals, USA, #NB100-1028), glial fibrillary acidic protein (1:2000; Abcam, USA, #ab7260), growth associated protein 43 (1:100; Abcam, USA, #ab75810) and synaptophysin (1:200; Abcam, USA, #ab52636) at 4 ℃ overnight. Then, the slides were washed with 0.01 M PBS three times and incubated with secondary antibodies (1:500; Jackson, USA, #711-545-152 or #705-585-147) at room temperature away from light. A fluorescence microscope (Leica, Germany, DM2500) and confocal laser scanning microscope (Olympus, Japan, FV3000) were used to acquire images, and brightness, gain, and contrast were all kept constant during image acquisition. In two serial brain sections from each mouse, the GAP-43 and synaptophysin staining in four fields and Iba1-positive amoeboid microglia in three fields in the cortex surrounding the lesion, and glial scar area were acquired with the same parameters, respectively. All samples were stained and photographed at the same time using the same batch of reagents. Before puncta analysis of GAP-43 and Synaptophysin, all images of each index are imported into ImageJ software to set brightness, contrast and threshold values to a fixed range. Puncta were quantified using ‘Analyze Particles’ with ImageJ software. The glial scar region is around lesion area with an intensive expression of GFAP, and the glial scar area was calculated using ‘Measure’ with ImageJ software.

**8. Transcriptome analysis**

Total mRNA of the cortex tissue surrounding the lesion was extracted from the sham, cTBI, and DCPC groups (n=3) using TRIzol (Invitrogen, USA, #15596026). The mRNA was purified and fragmented into small pieces to construct a cDNA library. The library was amplified to make DNA nanoballs (DNBs), DNBs were loaded into the patterned nanoarray and single-end 50-bases reads were generated on BGIseq500 platform (BGI-Shenzhen, China) for sequencing. Fragments per kilobase per million mapped fragments (FPKM) was used to standardize and determine the expression levels for mRNAs. The DEGs were selected with |log2FoldChange|>1 and *P* value<0.01. The R ggplot2 package was used to plot the volcano plot between group. The R VennDiagram package was used to draw venn diagram. The heatmap was generated using the R pheatmap package. GO analysis was performed using the web-based tool Metascape (https://metascape.org/gp/index.html). KEGG pathway enrichment analysis was performed by the web-based tool KOBAS (http://kobas.cbi.pku.edu.cn/kobas3/genelist/). The PPI network was obtained from the STRING online database (https://string-db.org). The PPI pairs with a combined confidence score ≥ 0.9 were visualized through Cytoscape. The iRegulon plugin in Cytoscape was employed to predict transcription factors regulating DEGs.

**9. Quantitative Real-Time PCR**

Total mRNA of the cortex tissue surrounding the lesion of the cTBI and DCPC groups, and same area tissues of sham group was extracted using TRIzol (Sangon, China, #B511311). After the extraction of total mRNA, MonScript™ RTIII All-in-One Mix with dsDNase (Monad, China, #MR05101) was used to conduct the reverse transcription reaction and synthesize cDNA. RT‒PCR was performed on a CFX96TM Real-Time System (Bio-Rad Laboratories Ltd., USA) according to the Hieff UNICON qPCR SYBR Green Master Mix kit (YEASEN, China, #11195ES08) to detect the mRNA expression of genes. *Gapdh* was used as a reference to normalize gene expression. The primers were synthesized by biotechnology company (Sangon, China), and the sequences are listed in Supplementary Table 1.

**10. Western Blot analysis**

Protein samples were lysed with RIPA buffer, and the concentration was detected by a BCA kit (BOSTER, China, #AR0146). Protein samples were isolated by SDS‒PAGE and transferred to polyvinylidene fluoride membranes (Millipore, Germany, #ISEQ00010). The membranes were blocked with 5% skimmed milk at 37 ℃ for 1 h and incubated with primary antibodies against GAPDH (1:5000; Bioworld, China, #AP0063) or IRF7 (1:1000; CST, USA, #72073) at 4 ℃ overnight. Then, the membranes were incubated with HRP secondary antibodies (1:5000; Boster, China, #BA1050 and #BA1054). Finally, the blots were detected by a chemiluminescence imaging system (Bio-Rad, USA).

**11. Open field test**

We used normal mice that inhaled 10% CO_2_ (10’/10’ × 3/d, 10 min inhalation/10 min break for 3 times per day) for 14 consecutive days. Three hours after CO_2_ inhalation on Day 13, we performed an open field test with the animals fully awake. Mice were exposed to an open field box (40 × 40 × 40 cm) with a center zone in the middle of the box (20 × 20 cm) marked with permanent marker.
Each mouse was placed in a corner facing the center and allowed to move freely for 10 min. A camera was placed directly above it to record the activity trajectory of mice in the open field box. Smart3.0 software was used to record the mice's movements separately and assess their locomotor ability.

**12. The measurement of heart rate, blood oxygen and respiratory rate**

We used normal mice that inhaled 10% CO_2_ (10’/10’ × 3/d, 10 min inhalation/10 min break for 3 times per day) for 14 consecutive days. After performing open field test on Day 13, mice were anesthetized with isoflurane, and heart rate, blood oxygen and respiratory rate were measured by Small Animal Vital Signs Monitor (STARR Life Sciences, USA).

**13. The measurement of blood pressure**

We used normal mice that inhaled 10% CO_2_ (10’/10’ × 3/d, 10 min inhalation/10 min break for 3 times per day) for 14 consecutive days. Then, we measured the blood pressure of the mice three hours after CO_2_ inhalation on Day 14. The cuff of noninvasive blood pressure (Kent scientific, USA, CODA Monitor) was first preheated to 37°C, and then the animals were loaded into the cuff. After the animals were in a stable condition, their systolic and diastolic blood pressures were measured.

**Supplementary Figures**

**Supplementary Figure 1**

**
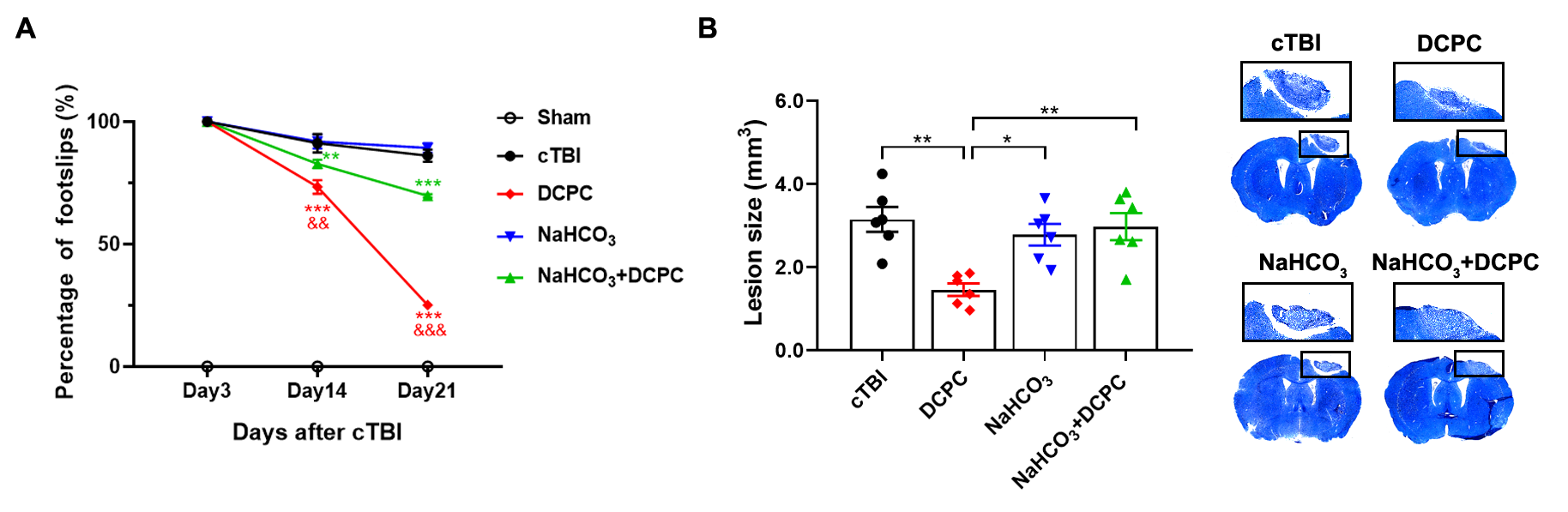
**

**Supplementary Figure 2**

**
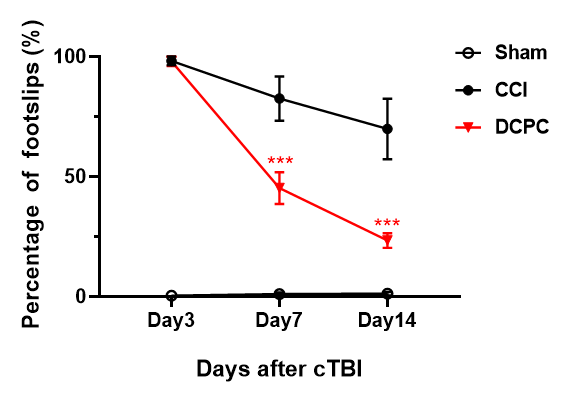
**

**Supplementary Figure 3**

**
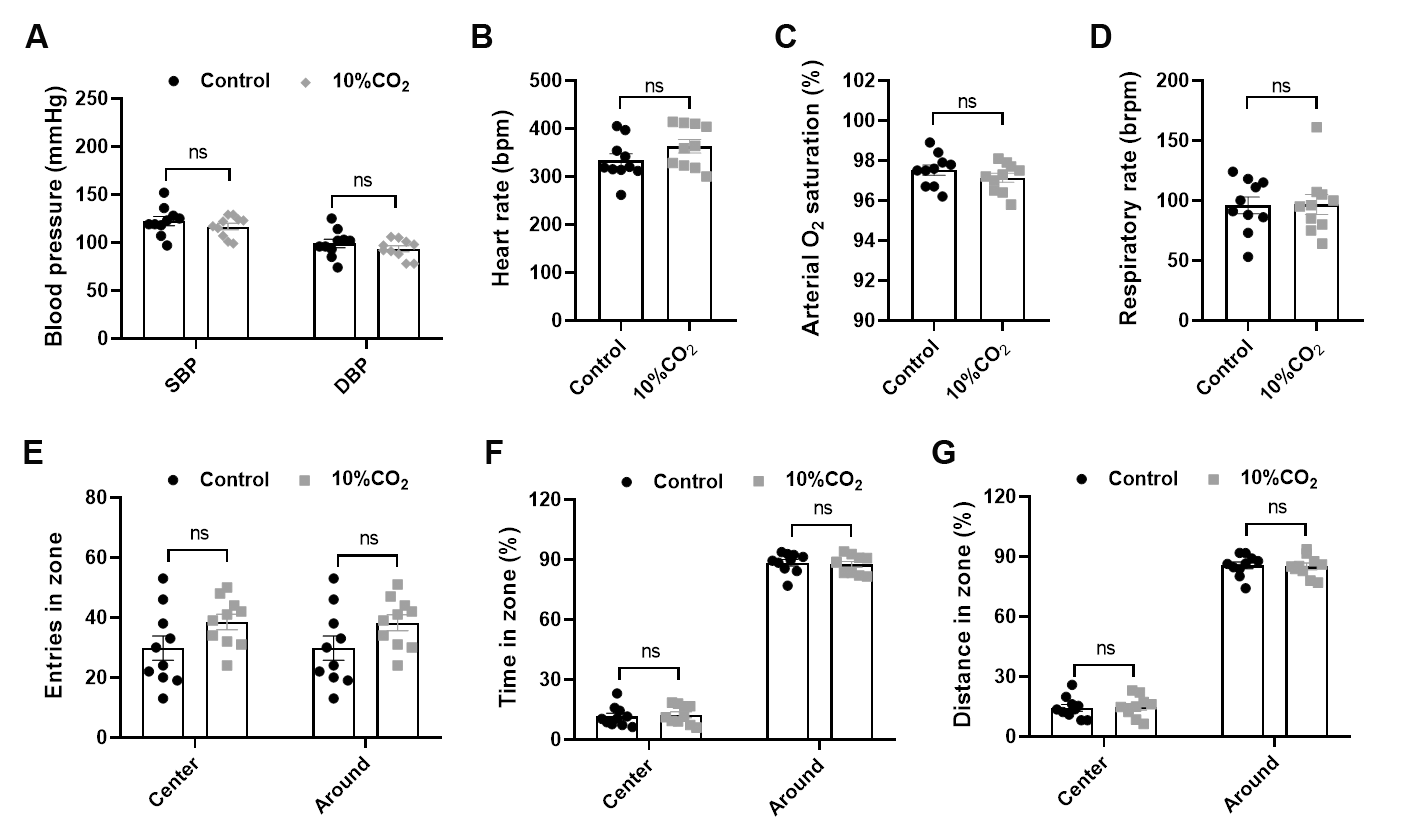
**

**Supplementary Figure legends**

**Supplementary Figure 1. Effects of NaHCO_3_ on DCPC-induced neuroprotection.** (A) Effect of DCPC (10% CO_2_, 10’/10’ × 3/d, administered at Days 3-14 after cTBI) and NaHCO_3_ on the percentage of footslips in the left hindlimbs, ***P*<0.01, ****P* < 0.001 vs. cTBI group, && *P*<0.01, &&& *P*<0.001 vs. NaHCO_3_ + DCPC group. (B) Effect of DCPC (10% CO_2_, 10’/10’ × 3/d, administered at Days 3-14 after cTBI) and NaHCO_3_ on lesion size at Day 14 post-injury, the right panel showed the representative coronal brain sections for lesion size, **P*<0.05, ***P*<0.01 vs DCPC group. Two-way ANOVA with Tukey’s test was used for analysis in A. One-way ANOVA with Tukey’s test was used for analysis in B. The results are shown as the mean ± SEM, n=6. 10’ /10’: 10-min CO_2_ inhalation/10-min break.

**Supplementary Figure 2.** **Effects of DCPC on motor function outcomes after CCI.**

Effect of DCPC (10% CO_2_, 10’/10’ × 3/d, administered at Days 3-14 after CCI) on the percentage of footslips in the left hindlimbs, ****P* < 0.001 vs. CCI group. Two-way ANOVA with Tukey’s test was used for analysis. The results are shown as the mean ± SEM, n=6. 10’ /10’: 10-min CO_2_ inhalation/10-min break.

**Supplementary Figure 3. Effects of DCPC on cardiovascular function and** **anxiety-related behavior.** Effect of DCPC (10% CO_2_, 10’/10’ × 3/d) on cardiovascular function including Blood pressure (A), Heart rate (B), Arterial O_2_ saturation (%) (C) and Respiratory rate (D) and on anxiety-like behavior including Entries in zone (E), Time in zone (%) (F) and Distance in zone (%) (G). Two-way ANOVA with Tukey’s test was used for analysis in A, E, F and G. Unpaired t test was used for analysis in B, C and D. The results are shown as the mean ± SEM, n=10.

**Supplementary Table**

Supplementary Table 1. PCR primer sequences

| Genes | Forward primer 5′-3′ | Reverse primer 5′-3′ |
| --- | --- | --- |
| *Irf7* | AGTGCTGTTTGGAGACTGGC | ACAGCCCAGGCCTTGAAGA |
| *Ccl2* | CCTGTCATGCTTCTGGGCC | CTCTCCAGCCTACTCATTGGGAT |
| *Ccl4* | CCCTGCAGTCCCAGCTCT | GGAAGTGGGAGGGTCAGAGC |
| *Ccl5* | GACTCTGAGACAGCACATGCATC | GGAGTGGTGTCCGAGCCA |
| *Ccl12* | AAGCAGAAGATTCACGTCCG | ATCCAGTATGGTCCTGAAGATCAC |
| *Ccr2* | GCTCATCTTTGCCATCATGATT | TCATTCCAAGAGTCTCTGTCAC |
| *Cxcl9* | TCGGACTTCACTCCAACACA | CCTTATCACTAGGGTTCCTCGAA |
| *Cxcl10* | TGAACCCAAGTGCTGCCGT | TCCCTATGGCCCTCATTCTCACT |
| *Cxcl13* | GCAACGCTGCTTCTCCTCC | CCGATCTATGATGTTTAGACCGACAAC |
| *Tlr2* | TGCTGGGCTGACTTCTCTCA | GATGGATGTCGCGGATCGAC |
| *Ifit3* | TTCTGAACTGCTCAGCCCAC | TTCCCGGTTGACCTCACTCA |
| *Zbp1* | GGCTCTGGGAATGACGACAG | CCTTCCTGACGTGAGTGGTA |
| *Cfb* | GCACAACATGGGTGGAAACC | CCCGACCCCAAACACATACA |
| *Ifi44* | TGGCCAATTCTTGTCTGGGT | GGGCTACTCACATGCCAACA |
| *Dhx58* | CCAAGAGGCATCTAGAGACGG | TGGCTCACCAGGTGTACCCTAT |
| *Fcgr4* | GGGTTCCGGATATCTGTGGTG | ACAGCCTTTTGGAGACCAGC |
| *Oasl1* | TGGGTGAACCCTTATGAACCC | TGTGGGTGTCGCAGAAGATT |
| *Rtp4* | TCTGCACTAGCTTGCTCACG | TGGCATGATTTCTCAGGGGG |
| *Igf1* | CCCTGGGTGACACTTCTGAC | CCCACAGATCTGGAGAAGGC |
| *Oas2* | CTTCATTCAAACCCGGCCCA | GGGCGTACTTTGGGGGTAAA |
| *Ifit1* | TTACAGCAACCATGGGAGAGAAT | TCAAGGAACTGGACCTGCTCT |
| *Usp18* | TCTGAAAACCCTGGAGGATGC | AGAACCTGTTTCCAAGGCGT |
| *Isg15* | TCTGACTGTGAGAGCAAGCAG | ACCTTTAGGTCCCAGGCCATT |
| *Cybb* | TTTCTCAGGGGTTCCAGTGC | CAATTGTGTGGATGGCGGTG |
| *Dhx60* | AGTGTGGCTCTTTTGCTCCA | ACTCGCACCACTTTTTCATTTT |
| *Gapdh* | GTCGGTGTGAACGGATTTGG | GCTCCTGGAAGATGGTGATGG |
