# Supplementary material for: Delayed CO2 postconditioning promotes neurological recovery after cryogenic traumatic brain injury by downregulating IRF7 expression
Source: CNS Neurosci Ther. 2023 May 19;29(11):3378–90. doi: 10.1111/cns.14268 (PMC10580333; doi:10.1111/cns.14268)

1. Figure 5B

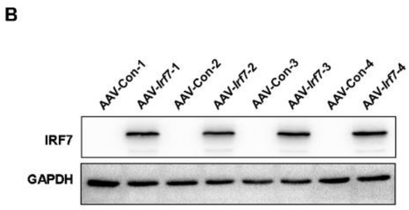

Full unedited gel/blot for Figure 5B.

Figure 5B: GAPDH

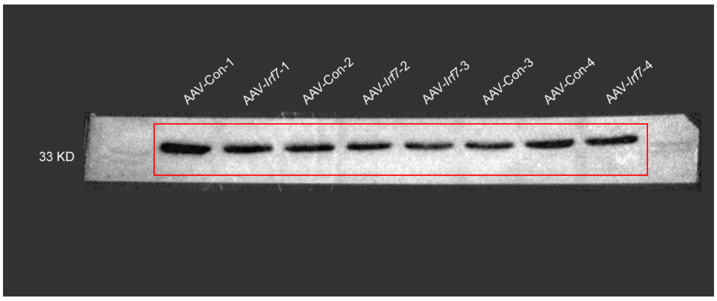

Figure 5B: IRF7

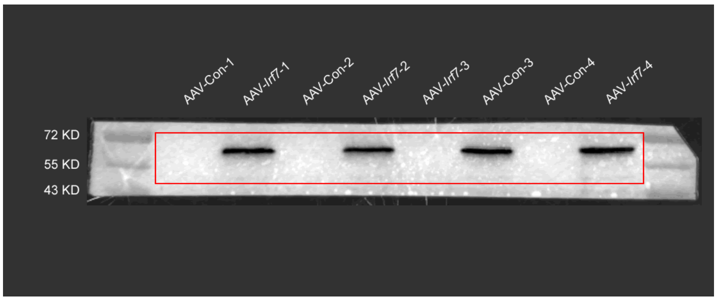

2. Figure 6A

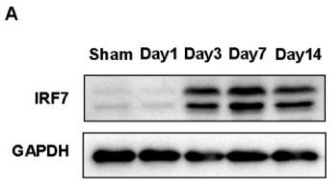

Full unedited gel/blot for Figure 6A.

Figure 6A: GAPDH

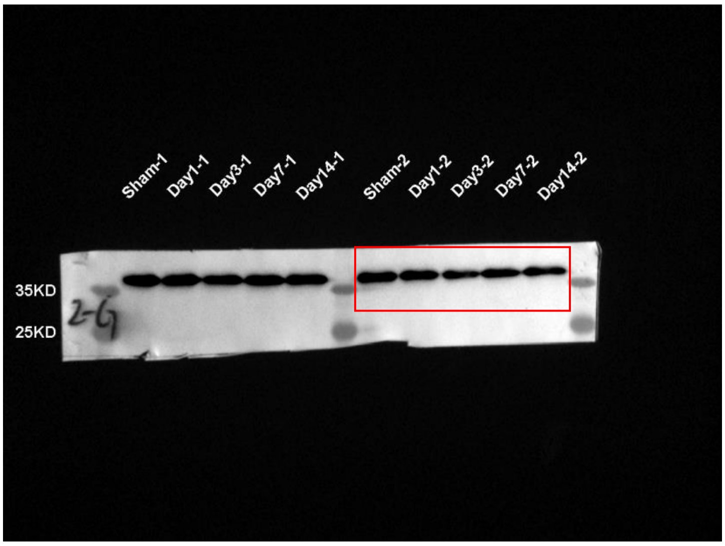

Figure 6A: IRF7

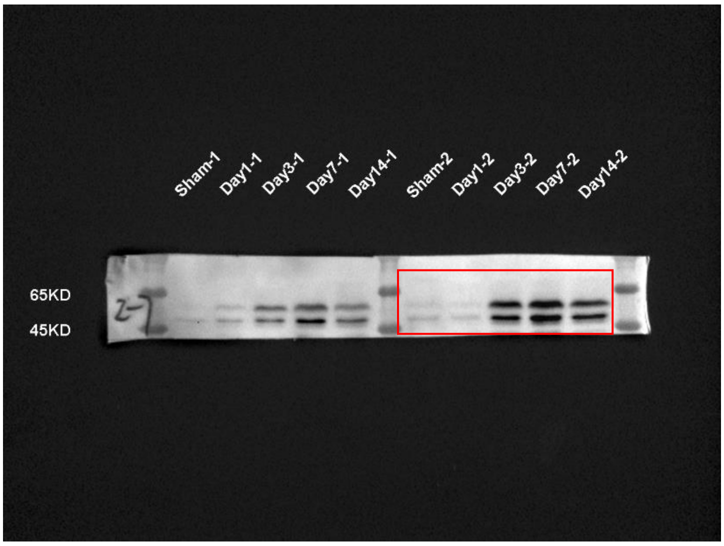

3. Figure 6B

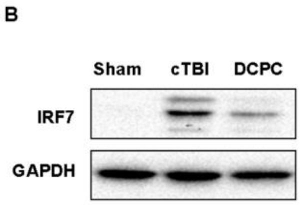

Full unedited gel/blot for Figure 6B.

Figure 6B: GAPDH

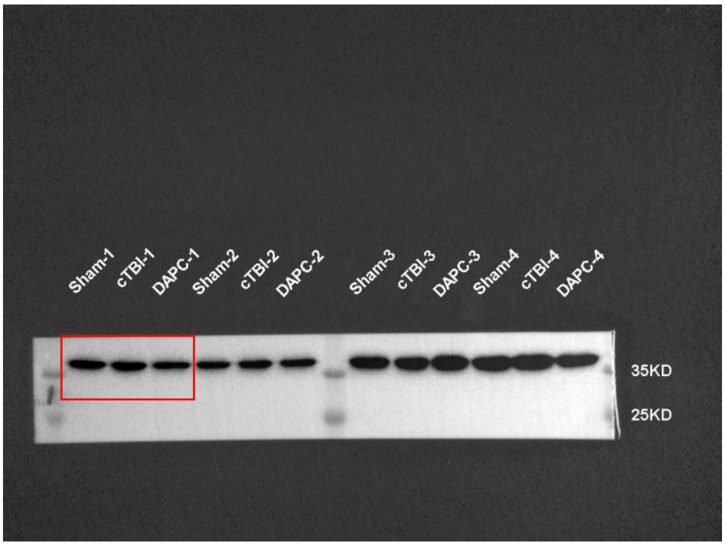

Figure 6B: IRF7

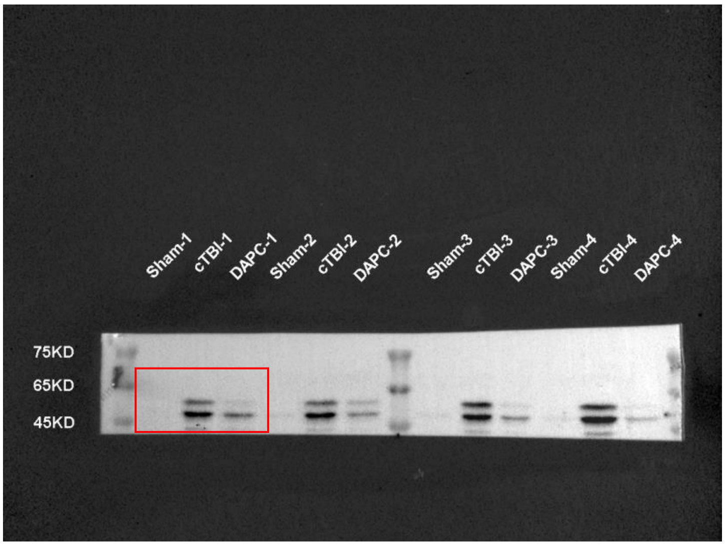

Supplement: Supplementary file 2 — Appendix S2. [file CNS-29-3378-s003.pdf]
